# Ruptured left ventricular pseudoaneurysm in the mediastinum following acute myocardial infarction: a case report

**DOI:** 10.1186/2047-783X-18-2

**Published:** 2013-01-21

**Authors:** Daoyuan Si, Kaiyao Shi, Dongmei Gao, Ping Yang

**Affiliations:** 1 Department of Cardiology, China-Japan Union Hospital of Jilin University, Changchun, 130021, China

**Keywords:** Aneurysm, Coronary disease, Myocardial infarction

## Abstract

Left ventricular pseudoaneurysm is an uncommon complication after transmural acute myocardial infarction (AMI). Here we describe the case of a 43-year-old man who presented with AMI and chest distress despite the normal appearance of his coronary artery during coronary angiography. Timely thrombolytic therapy was administered. Echocardiography, and cardiac computed tomography showed a ventricular pseudoaneurysm, and direct visualization at the time of surgery showed that it had ruptured in the mediastinum instead of the pericardium. The survival rate of patients with ventricular pseudoaneurysm rupture is low. The rupture of ventricular pseudoaneurysm in the mediastinum is rare; therefore, this case is noteworthy.

## Background

Left ventricular free wall rupture in myocardial infarction (MI) is often fatal, and only a few patients undergo operation. The cardiac rupture may be clinically undetected and lead to pseudoaneurysm. A left ventricular (LV) pseudoaneurysm is formed when cardiac rupture is contained by pericardium, organizing thrombus, and hematoma. It has been reported to occur mostly at the inferior segments of the left ventricle, following occlusion of the right coronary or left anterior descending branches [1]. Because of its rarity, the natural progression of pseudoaneurysm of the left ventricle is not well established. They are believed to have a poor prognosis because of a high probability of rupture. Here we report a case with LV pseudoaneurysm that occurred after a recent inferior MI and ruptured in the mediastinum.

## Case presentation

A 43-year-old man with history of prior myocardial infarction presented to our hospital with chest pain and shortness of breath; 8 months previously, the patient had been treated at the local hospital for ST segment elevation myocardial infarction with acute high lateral wall injury. He was treated with thrombolytic therapy. The patient was discharged without an echocardiogram and cardiac catherization due to limited resources, and he had not had a follow-up with a cardiologist because both doctors and the patient did not pay much attention to his condition as the symptoms had almost disappeared by then. Recently, our patient had been complaining of intermittent and slight chest pain, shortness of breath at rest, and frequent awakening due to chest distress at night for a duration of 2 weeks.

**Figure 1:**
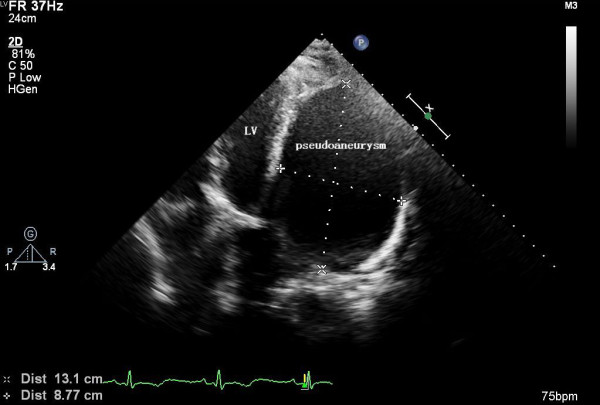
Preoperative echocardiography.

**Figure 2:**
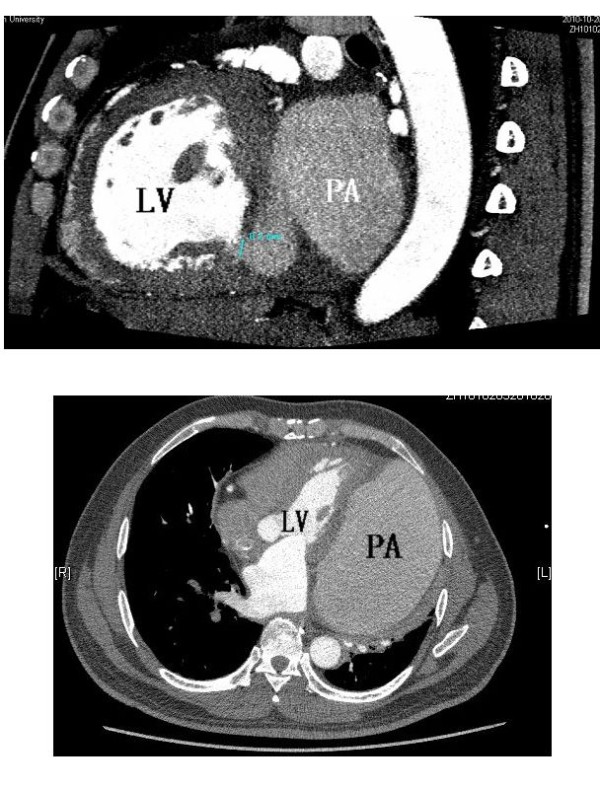
**Preoperative cardiac computed tomography.** PA, pseudoaneurysm.

Results of a physical examination indicated a blood pressure of 145/85 mm Hg, a heart rate of 88 beats/minute, a slightly expanded left margin of the heart, and a continuous 3/6 grade murmur between the third and fourth intercostal spaces at the left sternal border. An electrocardiogram examination revealed qR in leads I and aVL and inverted T-waves in leads I, II, aVL, and V_3_ to V_6_. Echocardiography showed a 13.1 × 8.8 cm limited oval anechoic area with a distinct boundary at the lateral posterior of the left ventricle, which was connected to the left ventricle at the basal segment of the lateral posterior wall of the left ventricle. Color Doppler sonography showed two streamlines of blood flow with a distance of 0.7 cm between the anechoic area and the left ventricle. Three-dimensional ultrasound indicated an echo dropout in an oval area in the basal segment of the lateral left ventricular wall, with a stroke volume (SV) of 70 mL, an ejection fraction (EF) of 63% and a cardiac output (CO) of 5.3 l/min. It indicated AMI and a perforation in the lateral wall of the left ventricle suggestive of a ventricular pseudoaneurysm (Figure 1). An enhanced cardiac computed tomography (CT) scan showed a lateral posterior mass in the left ventricle with a size of approximately 8.4 × 14.2 × 33.1 cm. We considered that it was a hematoma that had ruptured in the mediastinum, and it was partially connected to the left ventricle via a 0.8-cm tear. The layer of pericardium was not clear, so we could not rule out pericardial rupture from the CT scan. The heart was slightly shifted to the right due to the compression (Figure 2).

Coronary angiography showed normal development of the left and right coronary arteries without significant stenosis in the coronary trunk or branches.

**Figure 3:**
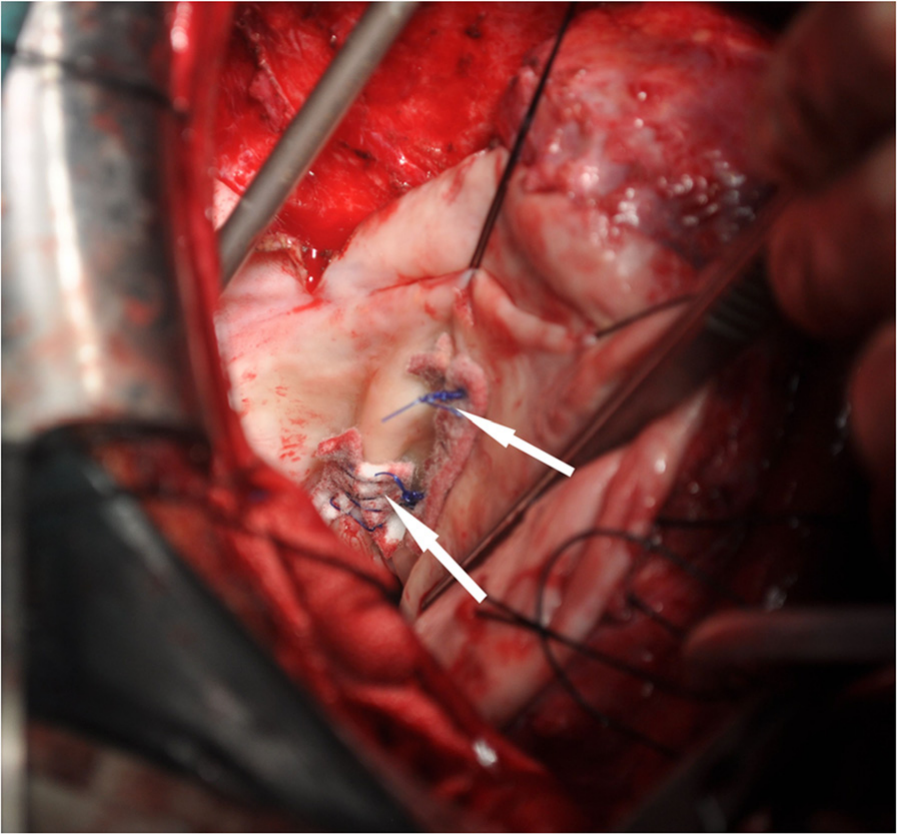
The two surgically repaired rupture sites.

The preoperative diagnosis was a ruptured ventricular pseudoaneurysm; thus, left ventricular aneurysm and perforation repair was the chosen treatment for this patient. However, intraoperative findings showed a pericardial rupture along the border of the infarct area and an approximately 8 × 13 × 30 cm cystic mass in the extrapericardial mediastinum in the left posterior side of the heart. The cystic wall adhered to the chest wall and was continuous with the left pleura. A hematocele and mural thrombus were found inside the incised cystic mass. Two transmural holes were revealed in the posterior wall of the left ventricle following removal of the hematocele and mural thrombus, the latter being connected to the left ventricular cavity (Figure 3). Pathological examination of the specimen (the left ventricular aneurysm wall) revealed thrombosis and hyperplastic fibrous tissues with hyaline degeneration but with no detectable hyperplasia or hyperemia in the covering epithelium or local blood vessels. The postoperative diagnosis was a ruptured ventricular pseudoaneurysm of the mediastinum.

Our patient was discharged 2 weeks later when his condition improved and he was symptom free at the follow-up visit. Follow-up echocardiography showed a 6.3 × 2.6 cm fluid sonolucent area outside the lateral wall of the left ventricle 1 year after the operation that was significantly smaller than it was at the previous examination and was considered a reactive exudation.

## Discussion

After AMI with the subsequent complication of ventricular pseudoaneurysm, patients are at risk for thrombus abscission, arrhythmia, and cardiac dysfunction. More importantly, the pseudoaneurysm body (epicardium) carries the risk of rupture at any time, resulting in cardiac tamponade. The rate of incidence of ventricular pseudoaneurysm ranges from 31% to 45% [2]. Cardiac rupture in the acute phase of AMI generally occurs within the first week after its onset and carries a 90% possibility of free wall rupture, which is associated with a high mortality rate [3]. In contrast, ventricular pseudoaneurysm rupture, which may occur during the recovery stage following AMI, is often induced by ischemia, fatigue, and emotional agitation and has a high mortality rate [4,5].

This is a rare case of ventricular pseudoaneurysm because the pseudoaneurysm rupture did not invade the pericardium or lead to pericardial tamponade and death. The clinical manifestations included only chest pain, shortness of breath, and frequent awakening due to chest distress at night, while normal cardiac function was seen using sonography. The key feature of this case was that the rupture involved the parietal pericardium and entered the mediastinum, which led to the formation of a cyst surrounded by the pleura. Due to the small rupture size, slow blood flow, and relatively large mediastinal space, the capacity and function of the right side of the heart were not affected. Rather, only manifestations associated with limited cardiac movement were observed, for example, chest tightness, shortness of breath, and awakening due to chest distress during the night, and no symptoms of pericardial tamponade were observed. As the disease progressed, the blood became organized in the mediastinum and formed a thrombus. The pericardium displayed hyaline degeneration and underwent adhesion with the pleura to become hardened tissue. Additionally, the presence of the oblique pericardial sinus inhibited the persistent and rapid expansion of the hematoma. Therefore, our patient did not die from acute blood loss.

Our patient, who suffered a transmural AMI, did not have a history of angina previously. No apparent stenosis was observed in the left or right coronary artery during coronary angiography. This observation indicates timely thrombolytic therapy and the recanalization of infarcted vessels. On the other hand, this observation also suggests that the patient did not experience chronic ischemia, thus providing no protection from collateral circulation in the area of the AMI. Consequently, during AMI, strong contraction of the cardiac muscles in the non-infarcted area may have an incising effect on the infarcted area that tended to favor the formation of a ventricular pseudoaneurysm or cardiac rupture [6].

The occurrence of pericardial tamponade has been prevented during the acute phase in some patients with free wall rupture of the left ventricle due to restriction from the thrombus at the rupture site and the parietal pericardium. However, the pseudoaneurysm wall, consisting of the organized thrombus and pericardial fibrous tissue, becomes unstable over time and may rupture at any time [7]. Our patient suffered a ventricular pseudoaneurysm rupture after an incident of AMI. Fortunately, the rupture entered the mediastinum, leading to the formation of a cyst encapsulated by the pleura, without the manifestation of pericardial tamponade symptoms or acute hemorrhagic shock. The existing literature reports that 30% to 45% of patients with ventricular pseudoaneurysm suffer cardiac rupture, with mortality rates of 48% after drug therapy and 23% after surgical treatment [8]. Therefore, it has been commonly accepted that ventricular pseudoaneurysms should be actively treated with surgical intervention once diagnosed and that conservative treatment is not recommended [9].

## Conclusions

Although our patient is fortunate that he did not die of ventricular pseudoaneurysm rupture, which usually leads to fatal pericardial tamponade, we understand that first, attention should be paid to the importance of following guidelines after AMI. Second, some of the basic tests such as echocardiograms and clinical follow-up visits with a cardiologist should not be neglected following improved symptoms, because the risk of cardiac rupture exists even for transmural myocardial infarction with a small infarcted area. Finally, once the formation of a ventricular pseudoaneurysm is detected, the site of damage should be surgically sutured as soon as possible to avoid the rupture of the ventricular pseudoaneurysm.

## Consent

Written informed consent was obtained from the patient for publication of this case report and any accompanying images. A copy of the written consent is available for review by the Editor-in-Chief of this journal.

## Competing interests

The authors declare that they have no conflict of interests.

## Authors’ contributions

DS and PY conceived and designed the experiments. DS performed the experiments. DS analyzed the data. KS and DGcontributed reagents/materials/analysis tools. DS wrote the manuscript. All authors read and approved the final manuscript.
